# Identification of two pathogenic guanidinoacetate methyltransferase variants in patients with intellectual disability: clinical and molecular insights

**DOI:** 10.1016/j.ymgmr.2026.101374

**Published:** 2026-10-17

**Authors:** Seyyed Reza Hashemi, AbdolAzim Nejatizadeh, Mohammad Hasan Sheikhha, Ali Reza Moayedi, Abbas Daneshipour, Shokouh Rajabi Firoozabadi, Seyed Ahmad Mohammadi, Seyed Mehdi Kalantar

**Affiliations:** a Department of Medical Genetics, Faculty of Medicine, Hormozgan University of Medical Sciences, Bandar Abbas, Iran; b Department of Medical Genetics, Faculty of Medicine, Shahid Sadoughi University of Medical Sciences, Yazd, Iran; c Pediatric Research Center, Hormozgan University of Medical Sciences, Bandar Abbas, Iran; d Abortion Research Center, Yazd Institute of Reproductive Sciences, Shahid Sadoughi University of Medical Sciences, Yazd, Iran; e Meybod Genetic Research Center, Meybod, Yazd, Iran

**Keywords:** Intellectual disability, *GAMT* deficiency, Whole-exome sequencing, Creatine deficiency syndromes, Guanidinoacetate, Genetic diagnosis

## Abstract

**Background:**

Intellectual disability (ID) is a genetically heterogeneous neurodevelopmental disorder with diverse underlying causes. Guanidinoacetate methyltransferase (GAMT) deficiency is a rare autosomal recessive disorder of creatine biosynthesis characterized by cerebral creatine depletion and accumulation of guanidinoacetate (GAA), resulting in a spectrum of neurodevelopmental manifestations, including intellectual disability. This study aimed to investigate the molecular basis of ID in individuals with suspected genetic etiologies and to characterize pathogenic *GAMT* variants.

**Methods:**

Whole-exome sequencing (WES) was performed in 50 unrelated Iranian individuals with idiopathic ID. Variants were prioritized based on population allele frequency, predicted functional impact, and genotype–phenotype correlation. Candidate variants were confirmed by Sanger sequencing, and segregation analysis was performed to establish their inheritance patterns. Biochemical and cerebral magnetic resonance spectroscopy findings were also evaluated in individuals with pathogenic *GAMT* variants.

**Results:**

Pathogenic biallelic *GAMT* variants were identified in two unrelated families. Patient 1 harbored the homozygous nonsense variant NM_000156.6:c.134G > A (p.Trp45Ter), whereas Patient 2 carried a novel homozygous four-base-pair deletion, NM_000156.6:c.373_376del (p.Pro125ThrfsTer35), predicted to result in premature protein truncation. Both variants were absent from the population databases examined and were classified as pathogenic according to ACMG/AMP criteria. Sanger sequencing confirmed homozygosity in the affected individuals and heterozygosity in their parents, consistent with autosomal recessive inheritance. Both patients exhibited global developmental delay, intellectual disability, severe speech impairment, behavioral/autistic features, and a history of seizures. Elevated plasma GAA concentrations and reduced cerebral creatine levels further supported the diagnosis of GAMT deficiency.

**Conclusions:**

This study expands the molecular spectrum of *GAMT*-related disease by identifying a novel pathogenic frameshift variant and provides additional clinical and biochemical characterization of GAMT deficiency in Iranian individuals with ID. The findings highlight the importance of considering GAMT deficiency among the potentially treatable causes of unexplained intellectual disability, particularly when neurodevelopmental manifestations are accompanied by biochemical evidence of disturbed creatine metabolism. Early molecular and biochemical recognition may facilitate appropriate clinical management and genetic counseling.

## 1 Introduction

Intellectual disability (ID) is a clinically and genetically heterogeneous neurodevelopmental disorder characterized by limitations in intellectual functioning and adaptive behavior with onset during the developmental period. Its phenotypic spectrum ranges from mild to severe impairment, and affected individuals may present with additional neurological and neurodevelopmental features, including developmental delay, epilepsy, speech and language impairment, behavioral abnormalities, and autism spectrum features [1], [2], [3], [4], [5]. The substantial genetic heterogeneity of ID includes chromosomal abnormalities, copy-number changes, single-gene disorders, and inherited metabolic conditions. Among these, cerebral creatine deficiency syndromes (CCDS) represent a clinically important group of metabolic disorders that can manifest with ID and other neurological abnormalities and, importantly, include potentially treatable conditions [6], [7].

Creatine is a major component of cellular energy metabolism and is particularly important in tissues with high and fluctuating energy requirements, including the brain and skeletal muscle. Endogenous creatine biosynthesis occurs through a two-step pathway. Arginine:glycine amidinotransferase (AGAT), encoded by *GATM*, catalyzes the formation of guanidinoacetate (GAA) from arginine and glycine. GAA is subsequently methylated by guanidinoacetate methyltransferase (GAMT), encoded by *GAMT*, using S-adenosylmethionine to generate creatine [8]. Disruption of this pathway can lead to cerebral creatine deficiency and neurodevelopmental disease.

Guanidinoacetate methyltransferase deficiency (GAMT deficiency; OMIM 601240) is an autosomal recessive CCDS caused by pathogenic variants in *GAMT*. Impaired GAMT activity blocks the final step of endogenous creatine synthesis, resulting in reduced creatine availability and accumulation of GAA. The resulting cerebral creatine depletion and increased GAA concentrations are associated with a characteristic but variable neurological phenotype, commonly including global developmental delay, ID, severe speech impairment, epilepsy, and behavioral or autistic features [9], [10]. This clinical variability may complicate recognition of GAMT deficiency, particularly when patients initially present with nonspecific developmental or behavioral abnormalities.

The diagnosis of GAMT deficiency is particularly relevant because it is a potentially treatable metabolic disorder. Increased GAA concentrations and reduced cerebral creatine provide important biochemical clues, whereas molecular analysis can establish the underlying genetic diagnosis and confirm the autosomal recessive inheritance pattern. The availability of biochemical and molecular approaches also emphasizes the importance of early recognition, as timely diagnosis may facilitate appropriate metabolic management, clinical monitoring, and genetic counseling [11]. Recent newborn screening studies have further demonstrated the feasibility of identifying affected individuals before the full neurological phenotype becomes apparent, supporting the value of early biochemical and molecular recognition of the disorder.

Despite the diagnostic importance of GAMT deficiency, its clinical manifestations overlap with those of numerous genetic causes of ID. Consequently, a specific metabolic diagnosis may not be suspected at the initial clinical assessment. Advances in genomic technologies, particularly whole-exome sequencing (WES), have substantially expanded the ability to identify pathogenic variants responsible for unexplained neurodevelopmental disorders. WES enables simultaneous assessment of a large proportion of protein-coding genes and can identify rare pathogenic variants across diverse disease categories [12], [13]. Its diagnostic utility has been demonstrated in individuals with developmental disorders and ID, while analysis of familial samples can further assist in determining inheritance and variant segregation [14]. Accordingly, exome sequencing has become an important diagnostic approach for individuals with unexplained neurodevelopmental disorders [15].

In this context, the relationship between ID and GAMT deficiency is primarily diagnostic: ID constitutes a heterogeneous presenting phenotype, whereas GAMT deficiency represents a specific genetic and metabolic cause that can be established through complementary molecular and biochemical evaluation. WES can identify pathogenic *GAMT* variants even when GAMT deficiency has not been clinically suspected, while biochemical assessment can provide functional evidence of disrupted creatine metabolism and support interpretation of genetic findings.

Therefore, the present study investigated the molecular basis of unexplained ID in 50 unrelated Iranian individuals younger than 18 years who underwent WES. Two unrelated patients were identified with biallelic pathogenic *GAMT* variants. The variants were confirmed by Sanger sequencing and familial segregation analysis, and the associated clinical and biochemical findings, including plasma GAA concentrations and cerebral creatine levels, were characterized. By integrating molecular, clinical, and biochemical findings, this study aims to further characterize *GAMT*-related disease and contribute to the recognition of GAMT deficiency as a potentially treatable cause of unexplained ID.

## 2 Materials and methods

### 2.1 Ethical compliance and informed consent

The study was approved by the Ethics Committee of Hormozgan University of Medical Sciences, Bandar Abbas, Iran (IR.HUMS.REC.1404.059) according to the principles outlined in the Declaration of Helsinki. Before participation, comprehensive counseling was provided to all families regarding the scope and limitations of WES testing. Informed written consent was obtained from all adult participants. For participants under 18 years, written consent was secured from their legal guardians. The consent protocol explicitly included permission for the publication of de-identified clinical photographs and anonymized genomic data for scientific purposes.

### 2.2 Clinical recruitment and phenotypic characterization

A cohort of fifty (*n* = 50) unrelated individuals with a clinical diagnosis of intellectual disability (ID), all aged <18 years, was recruited through specialized neurodevelopmental clinics. Participants were evaluated for unexplained or idiopathic ID and were not selected based on a prior molecular diagnosis of guanidinoacetate methyltransferase (GAMT) deficiency. Whole-exome sequencing (WES) was performed for all 50 participants as part of the genetic investigation of the underlying causes of ID within the study cohort. WES was conducted in the context of this research study and should not be interpreted as indicating that WES was routinely available as a standard clinical diagnostic test at our institution.

Phenotypic characterization was conducted using a multidisciplinary approach, including neuropsychological assessment with standardized cognitive testing and evaluation of adaptive functioning in accordance with DSM-5 criteria. Comprehensive medical histories, two-generation pedigrees, and detailed physical examinations were obtained to characterize the clinical phenotype.

WES analysis identified biallelic pathogenic *GAMT* variants in two unrelated individuals from the cohort. These two individuals constitute the cases described in detail in the present study. The remaining 48 participants were not included in the detailed clinical, biochemical, and molecular characterization presented here because the present report specifically focuses on the identification and characterization of *GAMT*-related disease.

### 2.3 Genomic DNA extraction and quality control

Peripheral venous blood samples (5–10 mL) were collected in EDTA-coated vacuum tubes. Genomic DNA (gDNA) was isolated from the leukocyte-rich buffy coat using the SinaPure DNA Extraction Kit (SinaClon, Tehran, Iran), following the manufacturer's optimized protocols. To ensure the technical success of downstream sequencing, DNA concentration was quantified using the Qubit 4.0 Fluorometer (Thermo Fisher Scientific, USA). Purity was assessed via spectrophotometric analysis (NanoDrop 2000), accepting only samples with an A260/A280 ratio of 1.8–2.0. DNA integrity was verified by 1% agarose gel electrophoresis to confirm the absence of significant degradation.

### 2.4 Whole-exome sequencing (WES)

Library preparation and target enrichment were performed using the Agilent SureSelect Human All Exon V7 Kit (Agilent Technologies, Santa Clara, CA). Genomic DNA was fragmented using a Covaris S220 Focused-Ultrasonicator. Sequencing was performed on an Illumina platform. Whole-exome sequencing data were subjected to rigorous quality control assessment to ensure sequencing reliability and reproducibility. Sequencing generated an average on-target depth of 105×, with **97.2%** of target bases covered at ≥20×**.** Sequencing reads were aligned to the human reference genome (GRCh37/hg19) using the Burrows–Wheeler Aligner (BWA-MEM), achieving an overall mapping rate of 99.1%**.** Sequencing quality was assessed using standard metrics, including a mean Q30 score of 92.4%. Variant calling for single-nucleotide variants (SNVs) and small insertions/deletions (indels) was performed using the Genome Analysis Toolkit (GATK v4.0.2) HaplotypeCaller. Variants failing sequencing quality thresholds (QUAL <30, read depth < 10, or genotype quality <20) were excluded before downstream analyses. All samples passed the predefined quality control criteria.

### 2.5 Bioinformatics pipeline, variant filtering, and pathogenicity assessment

A multi-tiered filtering and prioritization strategy was implemented to identify causative variants from the raw exome sequencing data. Initially, common variants with a minor allele frequency (MAF) ≥0.01 in public population databases, including gnomAD (v2.1.1) and the 1000 Genomes Project, were excluded.

Candidate variants were subsequently prioritized using a curated gene panel associated with neurodevelopmental disorders (NDDs) and metabolic encephalopathies, followed by genome-wide evaluation to identify potential novel or unexpected disease-causing variants.

Given that the identified variants in this study were protein-truncating variants (PTVs), including nonsense and frameshift mutations, pathogenicity assessment focused on tools specifically relevant to loss-of-function variants. MutationTaster2021 was used to predict the functional consequences of the variants, while NMDEscPredictor was employed to evaluate the likelihood of nonsense-mediated mRNA decay (NMD). The Combined Annotation Dependent Depletion (CADD v1.6) score was used as an additional measure of deleteriousness, with a Phred-scaled score ≥20 considered supportive of pathogenicity. Evolutionary conservation of the affected genomic regions was assessed using GERP++ and PhyloP scores obtained from the UCSC Genome Browser.

Final variant interpretation and classification were performed according to the recommendations of the American College of Medical Genetics and Genomics and the Association for Molecular Pathology (ACMG/AMP). Initial automated classification was generated using InterVar and subsequently refined through manual curation, incorporating population frequency, segregation data, inheritance pattern, and genotype–phenotype correlation.

### 2.6 Orthogonal validation and segregation analysis

To confirm the technical accuracy of the findings obtained via WES, all putative disease-causing variants were validated using Sanger sequencing. Locus-specific primers flanking the candidate variants were designed using the Primer3 software. Sequencing reactions were performed on the Applied Biosystems 3500 Genetic Analyzer (Thermo Fisher Scientific). This orthogonal validation step verified the presence of the variants, determined their zygosity status, and facilitated familial segregation analysis to confirm the inheritance pattern within the affected pedigrees.

### 2.7 Biochemical analysis and neuroimaging assessment

#### 2.7.1 Plasma guanidinoacetate (GAA) measurement

Plasma guanidinoacetate (GAA) concentrations were measured by liquid chromatography–tandem mass spectrometry (LC–MS/MS) in an accredited metabolic laboratory. The laboratory reference interval for plasma GAA was 0.5–3.0 μmol/L. Plasma GAA concentrations were interpreted in conjunction with the clinical phenotype and molecular genetic findings to support the diagnosis of guanidinoacetate methyltransferase (GAMT) deficiency.

#### 2.7.2 Cerebral creatine assessment by ^1H-MRS

Cerebral creatine was assessed using proton magnetic resonance spectroscopy (^1H-MRS) as part of the neuroradiological evaluation. Quantitative cerebral creatine values were obtained from the MRS assessment and reported in mmol/L according to the quantitative analysis provided by the reporting center. Because quantitative metabolite measurements obtained by ^1H-MRS may vary according to the acquisition protocol, brain region analyzed, tissue composition, spectral-processing procedures, and calibration or reference method, the reported cerebral creatine values were interpreted according to the methodology and reference values used by the reporting center. Accordingly, the values were not considered against a universal ^1H-MRS reference interval.

## 3 Results

### 3.1 Clinical presentation

Whole-exome sequencing (WES) was performed in 50 unrelated Iranian individuals with idiopathic intellectual disability (ID). Two unrelated patients were identified as having biallelic pathogenic variants in the *GAMT* gene and were subsequently evaluated in greater clinical and biochemical detail. The remaining individuals in the cohort were not included in the present case-focused analysis.

#### 3.1.1 Patient 1

Patient 1 was a 14-year-old individual with a history of global developmental delay, intellectual disability, severe impairment of expressive and cognitive speech, and behavioral abnormalities. The neurobehavioral phenotype included repetitive hand-flapping, autistic behaviors, poor social interaction, hyperactivity, impulsivity, and emotional lability. Gross motor development was delayed. The patient also had a history of seizures; however, the available medical records did not provide sufficient information to further classify the seizure type, including age at onset, seizure semiology, or frequency.

Physical examination demonstrated microcephaly, with a head circumference below the third percentile (51 cm), short stature, and underweight status. The patient was reported to have been small for gestational age (SGA; birthweight <10th percentile). Neurological examination showed spasticity and increased deep tendon reflexes (+3), without clonus or a Babinski sign. Hypotonia was not observed.

Several facial and dental features were also noted, including arched eyebrows, esotropia, low-set ears, a convex/beaked nasal configuration, and widely spaced teeth. Cranial MRI and CT were reported as normal. The clinical and biochemical findings are summarized in Table 1.

**Table 1|:** Clinical features and detailed phenotype of the GAMT deficiency patients.

Clinical features	Case1	Case2	Typical feature of GAMT deficiency
Age(years)	14	17	
Birthweight	SGA (< 10%)	SGA (< 10%)	Variable
Present height	Short	Short	Variable
Present weight	Underweight	Underweight	Variable
Microcephaly	+, < 3 percentile (51 cm)	+, < 3 percentile (50 cm)	Yes
Arched eyebrows	+	+	Not consistently reported
Eyes	Strabismus (esotropia)	Strabismus(esotropia)	Variable
Ears	Low set	Low set	Not typical
Nasal root and tip	Convex and Beaked	Convex and Beaked	Not typical
Teeth	Widely spaced	Widely spaced	Not consistently reported
Spasticity	+	+	Yes
Touch hypersensitivity	+	−	Variable
Deep tendon reflexes (DTR)	+3 Clonus: − Babinski: −	+2 Clonus: − Babinski: −	
Gross motor delay	+	+	Yes
Hypotonia	−	−	Variable
Neuropsychiatric ID/LD^a^	Mild (IQ 50–70)	Mild (IQ 50–70)	Yes
Language delay	+	+	Yes
Repetitive behavior	Flapping hands	Flapping hands	Yes
Autistic behaviors	+	+	Yes
Poor social relationships	+	+	Yes
Hyperactivity	+	+	Yes
Impulsivity	+	+	Yes
Emotional lability	+	+	Variable
seizure	+	+	Yes
developmental delay	+	+	Yes
Cranial MRI	N	N	Variable (may be normal)
Cranial CT scan	N	N	Not characteristic
severe disturbance of expressive and cognitive speech	+	+	Yes
Plasma GAA (μmol/L)	12 μmol/L	14 μmol/L	Yes
Plasma GAA reference interval (μmol/L)	0.5–3.0	0.5–3.0	
Cerebral creatine by ^1H-MRS (mmol/L)	1.33 mmol/L	1.53 mmol/L	Yes
Urine GAA	−	N/A	N/A
Urine creatine	−	N/A	N/A
GAMT genotype	NM_000156.6.134G > A (p.Trp45Ter), homozygous	NM_000156.6.373_376del (p.Pro125ThrfsTer35), homozygous	
ACMG/AMP classification	Pathogenic	Pathogenic	
Parental segregation	Both parents heterozygous carriers	Both parents heterozygous carriers	

* The available medical records did not provide sufficient information to characterize seizure onset, semiology, or frequency.

GAA, guanidinoacetate; GAMT, guanidinoacetate methyltransferase; ^1H-MRS, proton magnetic resonance spectroscopy; N/A, not available; ACMG/AMP, American College of Medical Genetics and Genomics/Association for Molecular Pathology. Cerebral creatine values were obtained from quantitative ^1H-MRS and were interpreted according to the methodology and reference values used by the reporting center; no universal ^1H-MRS reference interval was applied.

a Intellectual disability/Learning disabilities.

#### 3.1.2 Patient 2

Patient 2 was a 17-year-old individual with global developmental delay, intellectual disability, severe expressive and cognitive speech impairment, and behavioral abnormalities. The patient exhibited repetitive hand-flapping, autistic behaviors, poor social interaction, hyperactivity, impulsivity, and emotional lability. Gross motor development was delayed. The patient also had a history of seizures; however, the available medical records did not provide sufficient information to further characterize the seizure type, age at onset, or frequency.

Physical examination showed microcephaly, with a head circumference below the third percentile (50 cm), short stature, and underweight status. The birthweight was within the SGA range (<10th percentile). Neurological examination demonstrated spasticity and increased deep tendon reflexes (+2), without clonus or a Babinski sign. Hypotonia was not observed.

The patient had several dysmorphic features, including arched eyebrows, esotropia, low-set ears, a convex/beaked nose, and widely spaced teeth. Touch hypersensitivity was not reported. Cranial MRI and CT were normal.

### 3.2 Biochemical and cerebral creatine findings

Biochemical evaluation demonstrated elevated plasma guanidinoacetate (GAA) concentrations in both patients. Plasma GAA concentrations were 12 μmol/L in Patient 1 and 14 μmol/L in Patient 2, compared with the laboratory reference interval of 0.5–3.0 μmol/L.

Cerebral creatine was evaluated by proton magnetic resonance spectroscopy (^1H-MRS). The measured cerebral creatine concentrations were 1.33 mmol/L in Patient 1 and 1.53 mmol/L in Patient 2, respectively, substantially below the reference range of 5–8 mmol/L used for interpretation. Thus, both patients demonstrated elevated plasma GAA together with markedly reduced cerebral creatine.

Urinary GAA and urinary creatine measurements were not available for either patient and therefore were not included in the biochemical assessment.

### 3.3 Molecular findings and familial segregation

#### 3.3.1 Patient 1: previously reported nonsense variant

WES identified a homozygous nonsense variant in exon 1 of *GAMT*, NM_000156.6:c.134G > A (p.Trp45*). This variant has previously been reported in individuals with GAMT deficiency. The variant was absent from the population databases evaluated, including gnomAD, the 1000 Genomes Project, ExAC, and Iranome (allele frequency = 0).

The c.134G > A substitution introduces a premature termination codon at amino acid position 45 and is predicted to result in a truncated GAMT protein. MutationTaster2021 predicted a disease-causing effect, while NMDEscPredictor indicated a high probability of nonsense-mediated mRNA decay. The variant had a CADD Phred-scaled score of 34.

According to the ACMG/AMP framework, the variant was classified as pathogenic based on the combined evidence of PVS1, PM2, and PP1. Sanger sequencing confirmed the homozygous state in the proband, whereas both clinically unaffected parents were heterozygous carriers, consistent with autosomal recessive inheritance (Fig. 1).

**Fig. 1:**
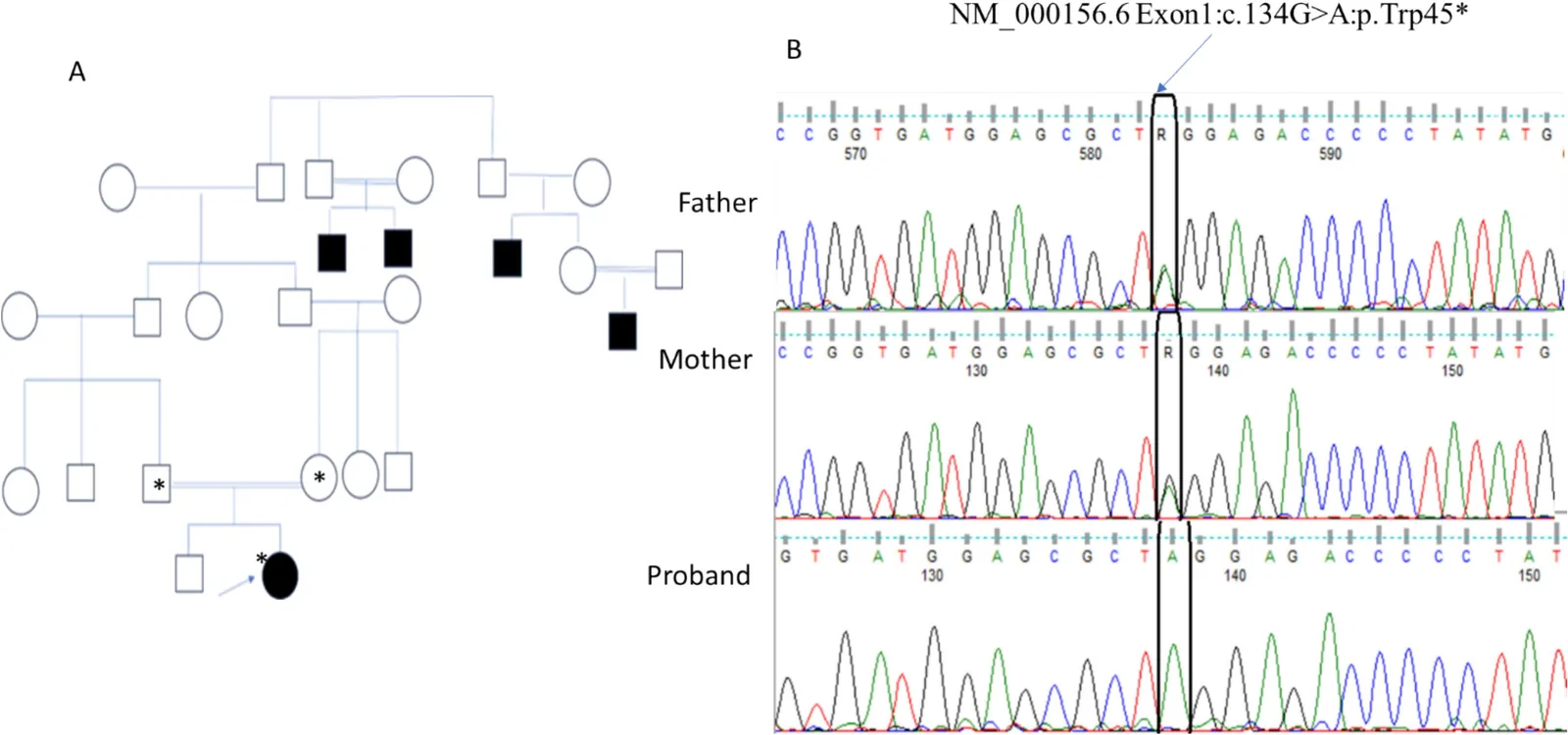
(A) Pedigree of Family 1 showing an autosomal recessive pattern of inheritance associated with intellectual disability. Individuals who underwent molecular analysis are indicated by an asterisk (*). The proband underwent whole-exome sequencing (WES), and the available parents underwent Sanger sequencing for segregation analysis. (B) Sanger sequencing confirmed the homozygous *GAMT* variant (NM_000156.6:c.134G > A) in the affected individual, while both parents were heterozygous carriers of the same variant.

#### 3.3.2 Patient 2: novel frameshift variant

In Patient 2, WES identified a homozygous four-base-pair deletion in *GAMT*, NM_000156.6:c.373_376del, predicted to result in p.Pro125ThrfsTer35. The variant was absent from gnomAD, the 1000 Genomes Project, ExAC, and Iranome (allele frequency = 0) and was not listed in ClinVar at the time of analysis. To the best of our knowledge, this variant has not previously been reported.

The deletion alters the reading frame beginning at codon 125 and introduces a premature termination codon 35 amino acids downstream, predicting substantial disruption of the GAMT protein. MutationTaster2021 predicted a disease-causing effect, and NMDEscPredictor supported a high probability of nonsense-mediated mRNA decay. The variant had a CADD Phred-scaled score of 35.

The variant was classified as pathogenic according to ACMG/AMP criteria based on PVS1, PM2, and PP1. Sanger sequencing confirmed the homozygous deletion in the affected individual, while both clinically unaffected parents were heterozygous carriers, confirming autosomal recessive segregation (Fig. 2).

**Fig. 2:**
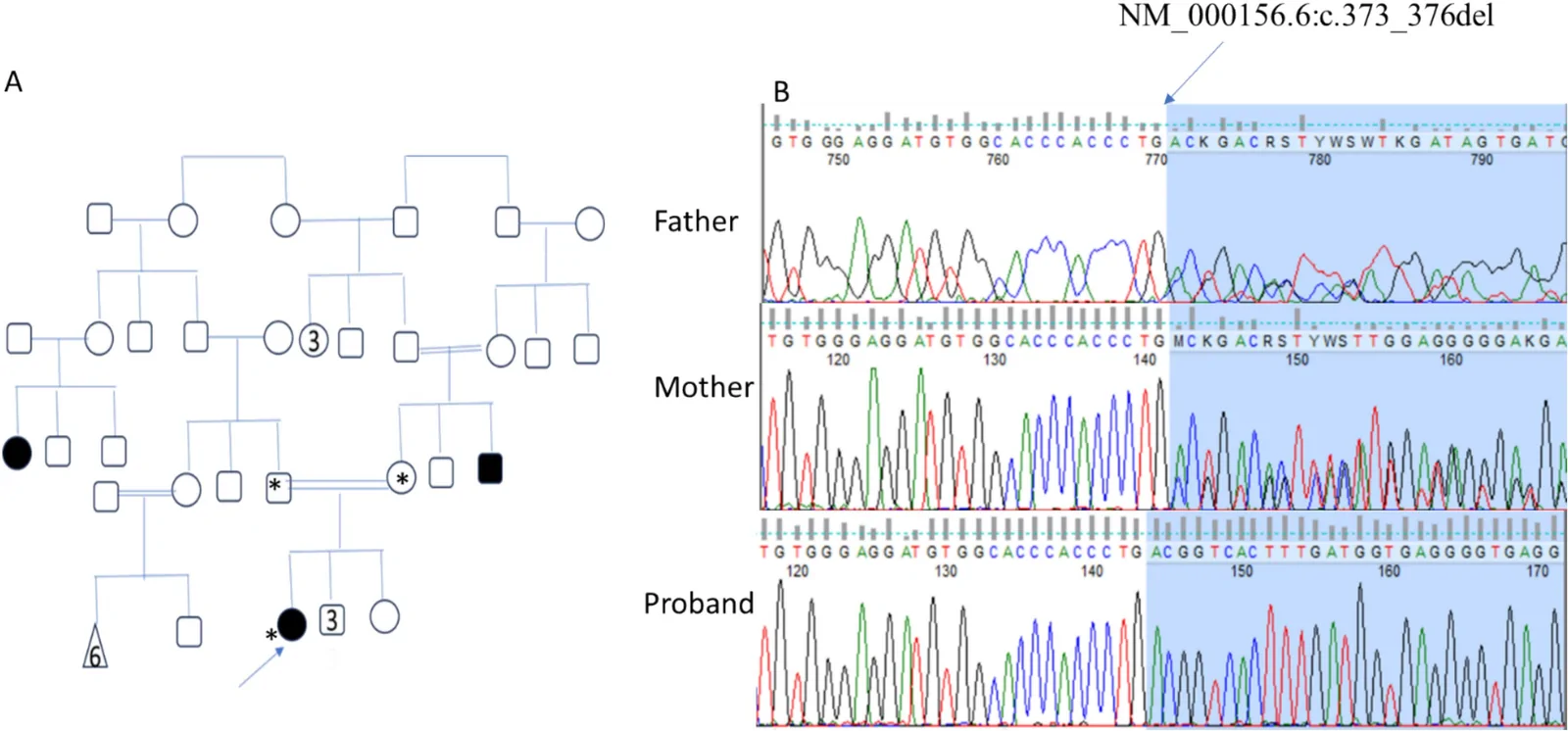
(A) Pedigree of Family 2 demonstrating an autosomal recessive pattern of inheritance. Individuals included in the molecular analyses are marked with an asterisk (*). Whole-exome sequencing was performed for the proband, whereas both parents were analyzed by Sanger sequencing to confirm segregation of the identified variant. (B) Electropherograms showing the *GAMT* variant (NM_000156.6:c.373_376del), detected in the homozygous state in the proband and in the heterozygous state in both parents, consistent with autosomal recessive inheritance.

Taken together, the two patients showed a consistent combination of neurodevelopmental impairment, elevated plasma GAA, markedly reduced cerebral creatine on ^1H-MRS, and biallelic pathogenic *GAMT* variants. Detailed treatment history and longitudinal follow-up data were unavailable; therefore, treatment response and long-term clinical outcomes could not be assessed.

## 4 Discussion

In the present study, whole-exome sequencing (WES) identified biallelic pathogenic variants in the *GAMT* gene in two unrelated individuals with intellectual disability (ID) and severe neurodevelopmental impairment. The identified variants comprised a previously reported nonsense variant, NM_000156.6:c.134G > A (p.Trp45*), and a novel four-base-pair deletion, NM_000156.6:c.373_376del (p.Pro125ThrfsTer35). Both variants were homozygous and consistent with the autosomal recessive inheritance of GAMT deficiency. The clinical manifestations, including global developmental delay, severe speech impairment, epilepsy, and behavioral abnormalities, were compatible with the established phenotypic spectrum of GAMT deficiency [16], [17], [18], [19].

Previous studies have demonstrated considerable clinical heterogeneity in GAMT deficiency. In the largest clinical series of 48 individuals, developmental delay/intellectual disability and epilepsy were major manifestations, while movement disorders were also reported [20]. Other studies have described severe developmental and speech impairment, epilepsy, behavioral or autism-spectrum abnormalities, and movement disorders [21], [22]. Thus, the neurological phenotype of our patients is broadly concordant with previously reported cases and supports the clinical relevance of the molecular findings.

The biochemical findings further supported the diagnosis. Both patients showed markedly elevated plasma guanidinoacetate (GAA) concentrations and reduced cerebral creatine levels on proton magnetic resonance spectroscopy (^1H-MRS). GAMT catalyzes the methylation of GAA to creatine; consequently, impaired GAMT activity results in GAA accumulation and reduced creatine synthesis [23], [24]. Elevated GAA in plasma or urine accompanied by reduced or low-normal creatine concentrations is an important biochemical pattern of GAMT deficiency [25], [26]. In our patients, the combination of elevated plasma GAA, reduced cerebral creatine, and biallelic pathogenic *GAMT* variants provided a coherent diagnostic framework.

^1H-MRS is a non-invasive method for detecting cerebral creatine deficiency and may demonstrate a markedly decreased or absent creatine signal in creatine deficiency syndromes [27], [28], [29]. Its diagnostic utility has also been specifically demonstrated in GAMT deficiency [30]. In the present patients, cerebral creatine concentrations were markedly below the reference interval, providing additional evidence of cerebral creatine depletion. The MRS findings therefore complemented the biochemical and molecular results.

Several clinical findings require cautious interpretation. Microcephaly should not be considered a novel feature because abnormal head size, including microcephaly, has been reported in individuals with GAMT deficiency [31]. Similarly, failure to thrive (FTT) has been described in individual patients [32]. Spasticity may also occur within the broader neurological spectrum of the disorder. Although hypotonia is frequently emphasized in creatine deficiency syndromes, spasticity and other abnormalities of muscle tone and movement have been reported, including in the 48-patient series [33]. Therefore, these findings may occur in GAMT deficiency but are neither specific nor newly established features.

The dysmorphic facial features observed in our patients, including arched eyebrows, low-set ears, a convex or beaked nose, and widely spaced teeth, are not considered classical manifestations of GAMT deficiency, whose recognized phenotype is predominantly neurological, developmental, behavioral, and movement-related [34]. Nevertheless, abnormal facial features have been described in individual patients [35]. Accordingly, these findings should be regarded as atypical and nonspecific. Similarly, small-for-gestational-age (SGA) status is not established as a characteristic feature in available clinical descriptions or larger patient series [36]. We therefore do not propose SGA as a newly recognized manifestation based on two patients. In consanguineous families, atypical findings may also raise the possibility of an additional genetic diagnosis, emphasizing the importance of comprehensive genomic interpretation [37].

The presence of atypical manifestations also illustrates the genetic heterogeneity of ID. Developmental delay, epilepsy, behavioral abnormalities, and abnormal muscle tone occur in numerous genetic neurodevelopmental disorders [38]. In the present study, WES was performed in 50 unrelated Iranian individuals with idiopathic ID, and two were found to have biallelic pathogenic *GAMT* variants. Although the remaining cohort was outside the scope of this report, the identification of GAMT deficiency among individuals initially investigated for unexplained ID demonstrates the value of broad genomic testing for uncovering rare metabolic disorders.

The choice of WES as the primary genomic approach also warrants consideration. Biochemical testing can provide a characteristic diagnostic signature of GAMT deficiency, particularly elevated GAA together with abnormalities in creatine metabolism [39]. When GAMT deficiency is specifically suspected, biochemical testing may therefore be an efficient initial diagnostic strategy. However, our patients were investigated because of idiopathic ID rather than a pre-established suspicion of GAMT deficiency. Given the marked genetic heterogeneity of ID, WES permits simultaneous evaluation of numerous neurodevelopmental genes rather than restricting analysis to *GAMT* [39]. Exome sequencing has consequently been supported as a first-tier diagnostic approach for unexplained neurodevelopmental disorders [40]. Our findings illustrate that WES can identify GAMT deficiency even when a specific metabolic disorder is not initially suspected.

The molecular findings further strengthen the diagnosis. The previously reported p.Trp45* variant introduces a premature termination codon and is predicted to result in loss of functional GAMT protein. The novel p.Pro125ThrfsTer35 variant is a four-base-pair deletion predicted to cause a frameshift and premature termination. The homozygous state, parental carrier status, segregation findings, protein-truncating nature, and concordant biochemical phenotype collectively support the pathogenicity of these variants. The identification of p.Pro125ThrfsTer35 therefore expands the known mutational spectrum of *GAMT* deficiency [41].

The differential diagnosis of cerebral creatine deficiency includes defects of creatine synthesis and transport, which may present with overlapping neurological manifestations [41]. X-linked creatine transporter deficiency, for example, may cause ID and neurological abnormalities and should be distinguished from defects of creatine biosynthesis [42]. In our patients, markedly elevated GAA, reduced cerebral creatine, and biallelic pathogenic *GAMT* variants supported GAMT deficiency rather than an isolated creatine transporter disorder.

Although GAMT deficiency is potentially treatable, detailed treatment discussion is beyond the scope of this study. Disease-specific therapeutic approaches aim to reduce GAA accumulation and restore creatine availability [43]. However, systematic treatment and longitudinal therapeutic follow-up were unavailable for our patients; therefore, treatment-response data cannot be reliably assessed. Establishing the diagnosis nevertheless remains clinically important because it enables appropriate genetic counseling and consideration of disease-specific management [24].

Several limitations should be acknowledged. Only two individuals with genetically confirmed GAMT deficiency were identified, limiting genotype–phenotype interpretation. Urinary GAA and creatine measurements were unavailable, and biochemical assessment was therefore based on plasma GAA and cerebral creatine measurements. Detailed seizure phenotyping and longitudinal treatment-response information were also unavailable. In addition, original ^1H-MRS spectral images were not available for inclusion as a figure. These limitations are particularly relevant when interpreting atypical findings such as SGA, dysmorphic features, microcephaly, FTT, and spasticity.

Overall, this study expands the clinical and molecular characterization of GAMT deficiency through the identification of two biallelic pathogenic variants in unrelated individuals with ID. The combination of compatible neurodevelopmental manifestations, elevated plasma GAA, reduced cerebral creatine on ^1H-MRS, and pathogenic *GAMT* variants provided a consistent diagnostic framework. The novel p.Pro125ThrfsTer35 frameshift variant expands the mutational spectrum of GAMT deficiency, whereas the atypical clinical findings should be interpreted cautiously and not automatically attributed to *GAMT* deficiency. These findings support an integrated diagnostic strategy combining clinical assessment, biochemical testing, ^1H-MRS, and genomic sequencing in individuals with unexplained ID [44].

### 4.1 Limitations of the study

This study has several limitations. First, the relatively small number of patients limits the generalizability of our findings. Second, although the identified variants were classified as pathogenic based on ACMG/AMP criteria and supported by multiple in silico predictions, functional assays were not performed to directly assess their impact on GAMT enzymatic activity. Third, detailed longitudinal follow-up and treatment response data were unavailable; therefore, the clinical outcomes following therapeutic interventions could not be evaluated. Future studies involving larger cohorts and functional characterization of novel GAMT variants are warranted to further elucidate genotype–phenotype correlations and improve clinical management of GAMT deficiency.

Although the identified variants were classified as pathogenic according to ACMG/AMP guidelines based on the combination of genetic, clinical, and computational evidence, functional assays were not performed to directly confirm their effects on GAMT protein function or enzymatic activity. Therefore, further experimental studies are required to provide additional functional evidence and better define the molecular consequences of these variants.

## 5 Conclusion

In this study, we identified one novel pathogenic variants in the *GAMT* gene in two unrelated Iranian families with ID. Our findings expand the mutational spectrum of GAMT deficiency and further support the value of WES for the diagnosis of treatable neurometabolic disorders. Because early diagnosis followed by appropriate metabolic therapy may substantially improve neurological outcomes, GAMT deficiency should be considered in the differential diagnosis of patients presenting with unexplained developmental delay, epilepsy, and severe speech impairment.

## Author contribution declaration

SRH conceptualized the study, designed the research, collected data, performed the analysis, and drafted the manuscript. AN served as principal investigator and was responsible for review and editing. MHSH served as principal investigator and was responsible for review and editing. AM contributed to conceptualization and supervision. SMK contributed to conceptualization, supervision, methodology, validation, and review editing.AD analyzed whole-exome sequencing and genetic data. SRF provided the clinical sample and data. SAM performed molecular work. All authors read and approved the final version of the manuscript.

## CRediT authorship contribution statement

**Seyyed Reza Hashemi:** Writing – original draft, Funding acquisition, Conceptualization. **AbdolAzim Nejatizadeh:** Supervision. **Mohammad Hasan Sheikhha:** Supervision. **Ali Reza Moayedi:** Supervision. **Abbas Daneshipour:** Software. **Shokouh Rajabi Firoozabadi:** Data curation. **Seyed Ahmad Mohammadi:** Data curation. **Seyed Mehdi Kalantar:** Supervision.

## Funding declaration

The authors declare that no specific funding was received for this work.

## Declaration of competing interest

The authors declare that they have no competing interests.

## Data Availability

The data supporting the findings of this study are available within the article. Additional data may be available from the corresponding author upon reasonable request.
